# Supplementary material for: Genetic perturbation of PU.1 binding and chromatin looping at neutrophil enhancers associates with autoimmune disease
Source: Nat Commun. 2021 Apr 16;12:2298. doi: 10.1038/s41467-021-22548-8 (PMC8052402; doi:10.1038/s41467-021-22548-8)
Supplement: Supplementary file 1 — Supplemetary Information [file 41467_2021_22548_MOESM1_ESM.pdf]

SUPPLEMENTARY INFORMATION

Genetic perturbation of PU.1 binding and chromatin looping at neutrophil enhancers associates with autoimmune disease

Stephen Watt[1], Louella Vasquez[1,§], Klaudia Walter[1,§], Alice L. Mann[1,§], Kousik Kundu[1], Lu Chen[1,2,3], Ying Yan[1], Simone Ecker[4], Frances Burden[5,6], Samantha Farrow[5,6], Ben Farr[1], Valentina Iotchkova[1,7,8], Heather Elding[1], Daniel Mead[1], Manuel Tardaguila [1], Hannes Ponstingl[1], David Richardson[7], Avik Datta[7], Paul Flicek[7], Laura Clarke[7], Kate Downes[5,6], Tomi Pastinen[9], Peter Fraser[10,11], Mattia Frontini[5,6,12,*], Biola-Maria Javierre[10,13,*,#], Mikhail Spivakov[10,14,15,*,#], Nicole Soranzo[1,2,*,#]

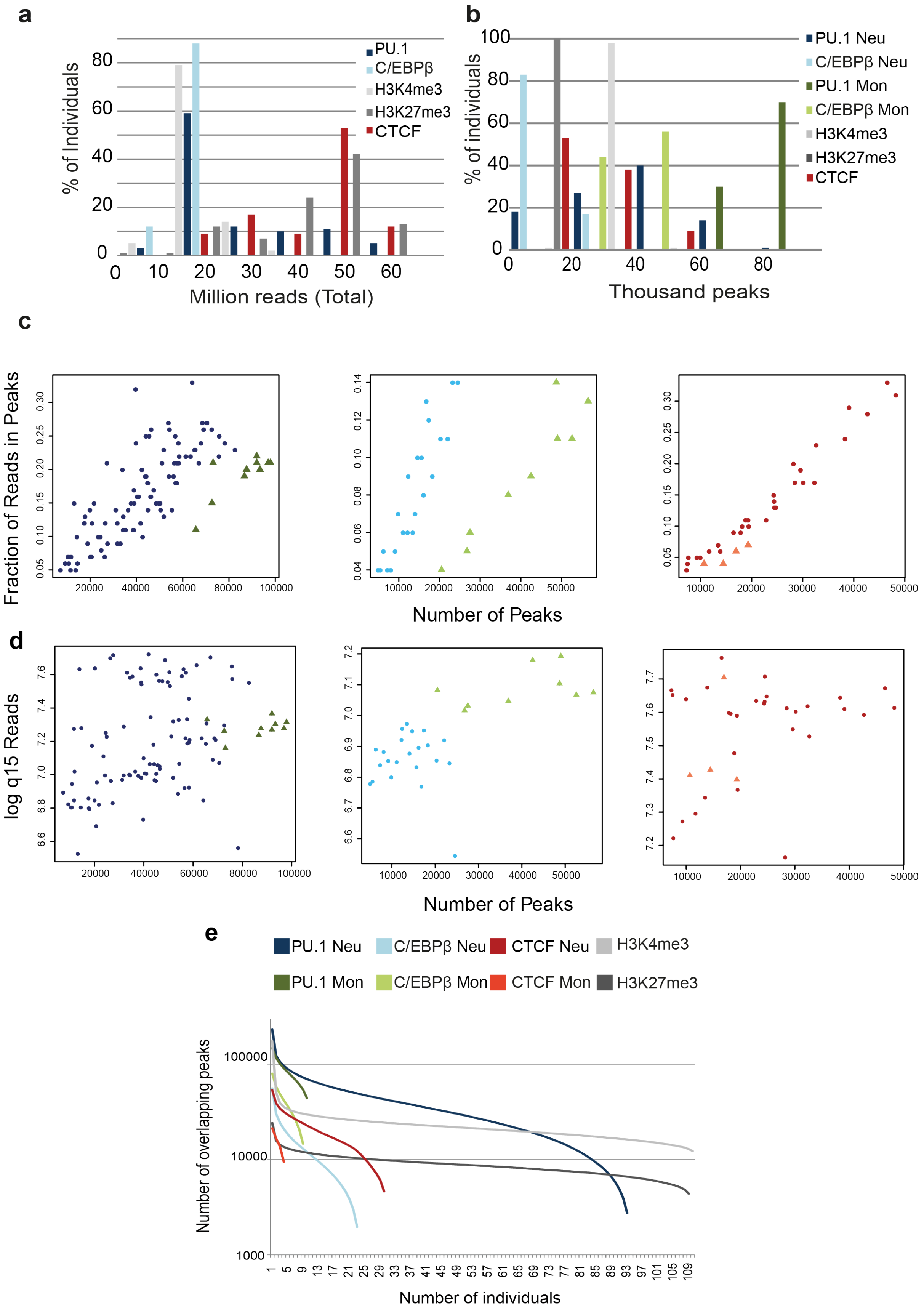

Supplementary Figure 1. Data QC plots for ChIP-seq data. a. Bar plot of bins showing the proportion of individuals with the total number of aligned reads for each factor probed. **b.** Bar plot of bins of number of peaks called for each transcription factor, individual and in the two cell types. Monocytes consistently

have more binding locations for both PU.1 and C/EBPβ over neutrophils data sets. **c.** (top) Scatter plot representing the fraction of QC passed (MAPQ >15) aligned reads (y-axis) that intersect the reference peak set, versus the number of peaks called for each ChIP-seq data set (x-axis). **d.** Scatter plot for the total number of QC passed (MAPQ >15) aligned reads (y-axis) versus the number of peaks called (x-axis). Neutrophil datasets are represented by dots and monocyte datasets by triangles. **e.** Peak overlap plot split by factor and cell type. y-axis represents the total number of peaks called in all individuals, x-axis represents the number of individuals where that peak is called.

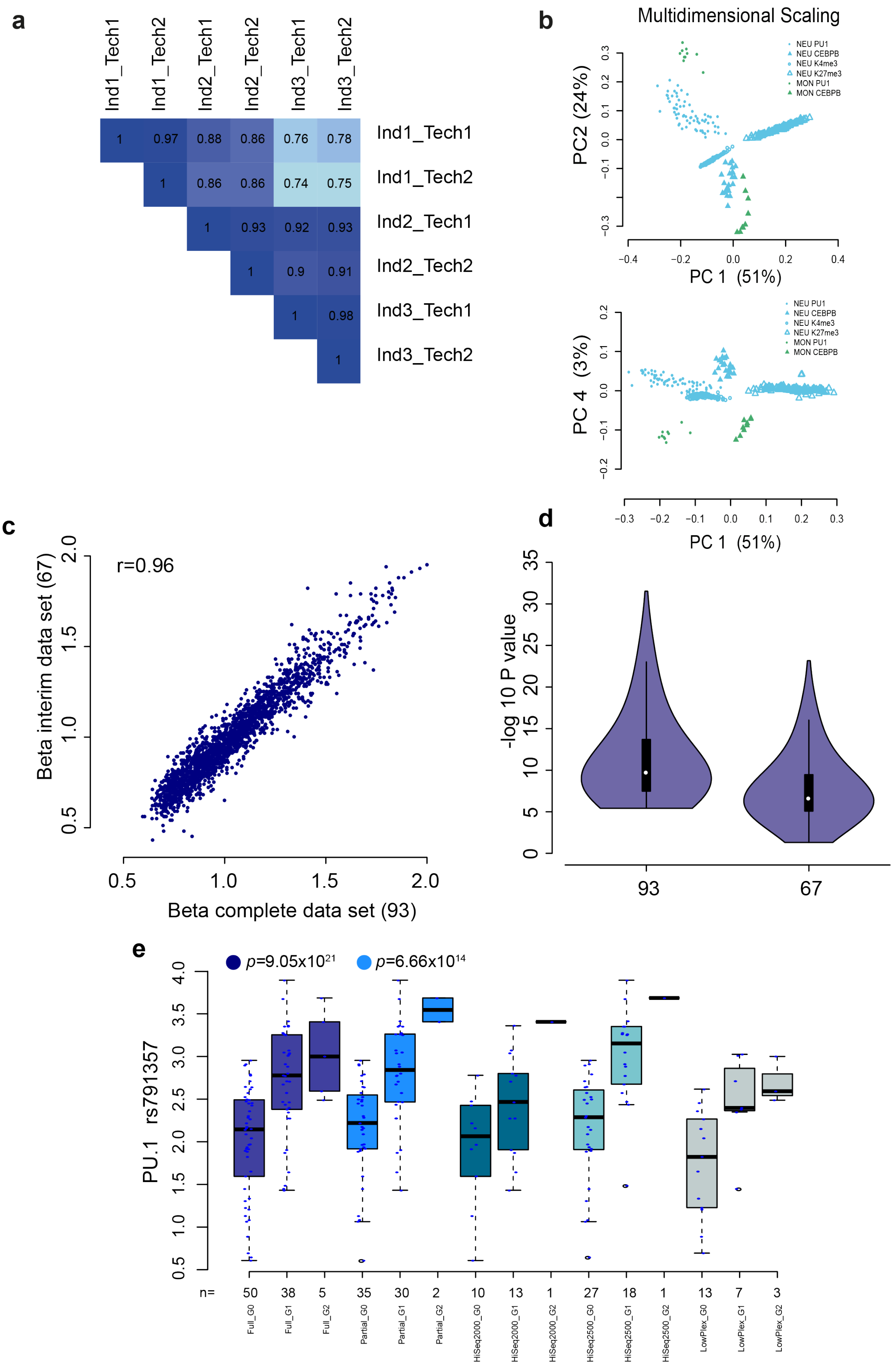

a
Ind1_Tech1
Ind1_Tech2
Ind2_Tech1
Ind2_Tech2
Ind3_Tech1
Ind3_Tech2
1 0.97 0.88 0.86 0.76 0.78
1 0.86 0.86 0.74 0.75
1 0.93 0.92 0.93
1 0.9 0.91
1 0.98
1
b
Multidimensional Scaling
NEU PU1
NEU CEBPB
NEU K4me3
NEU K27me3
MON PU1
MON CEBPB
PC2 (24%)
PC 1 (51%)
PC 4 (3%)
PC 1 (51%)
c
r=0.96
Beta interim data set (67)
Beta complete data set (93)
d
-log 10 P value
93
67
e
p=9.05x10^21
p=6.66x10^14
PU.1 rs791357
n=
50 38 5 35 30 2 10 13 1 27 18 1 13 7 3
Full_G0
Full_G1
Full_G2
Partial_G0
Partial_G1
Partial_G2
HiSeq2000_G0
HiSeq2000_G1
HiSeq2000_G2
HiSeq2500_G0
HiSeq2500_G1
HiSeq2500_G2
LowPlex_G0
LowPlex_G1
LowPlex_G2

Supplementary Figure 2. Data QC plots for ChIP-seq and tfQTLs. a. For three individuals, PU.1 profiling was performed in duplicate as technical replicates. Heatmap of pairwise analysis of logRPM (reads per million mapped) signal within a consensus peak set of ~55,000 shared sites, numbers are the Pearson's correlation between replicates. **b.** Variation in data sets shown by multidimensional scaling PC1 *versus* PC2 and PC1 versus PC4. **c.** Data was collected in three main tranches over a 24 month period. First on a HiSeq2000 at a high plex level, second moving to HiSeq2500, and thirdly on HiSeq2500 for both PU.1 and CTCF at increased depth, with the aim of performing combined haplotype or allele specific analysis as a validation set of the original smaller (67) tfQTL data set. Ultimately we proceeded with both QTL and CHT (Rasqual) testing on the total 93 donors collected. Presented are comparisons of the small and final PU.1 tfQTL datasets. Scatter plot showing the effect size (beta) between 2016 tfQTL features tested in both the interim QTL analysis and the fully acquired dataset, Pearson correlation is shown in the upper left of the plot. **d.** Violin plot displaying the *p*-value distribution for the significant tfQTLS from the full QTL dataset and the corresponding *p*-values obtained from interim dataset. Adding more donors increased the power to detect QTLs, yielding an additional 642 PU.1 QTLs passing the significance threshold. **e.** An illustrative example for PU.1 tfQTL rs791357 from Figure 5c, for both the full tfQTL data set and the interim data set with associated *p*-values from the QTL test. The same tfQTL split by the three batches, those which were run on HiSeq2000, HiSeq2500 and the low plex run also on HiSeq2500. Box plots show the medians (centre lines) and the twenty-fifth and seventy-fifth percentiles (box edges), with whiskers extending to 1.5 times the interquartile range. *p*-value were obtained by fitting linear mixed models implemented in LIMIX. n= the number of individual donors.

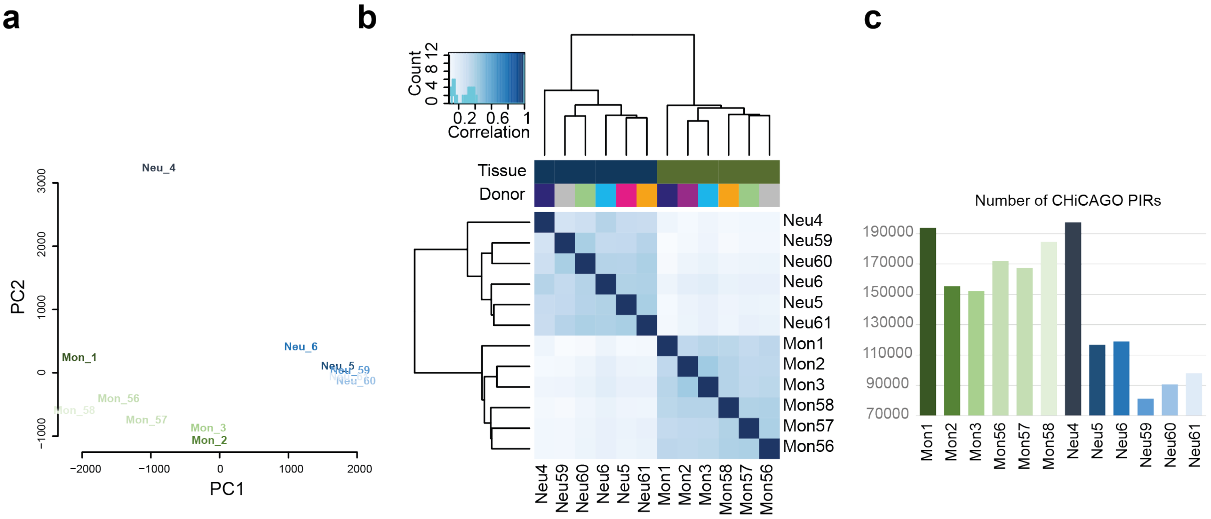

Supplementary Figure 3. Summary of PCHi-C data in two cell types. a. Principal component analysis plot showing first 2 principal components across twelve PCHi-C data sets. **b.** Heat map of Pearson correlations for intersecting (1bp overlap) PIRs (CHiCAGO >5) from twelve data sets. **c.** Bar plot of the number of promoter enhancer connections called with a CHiCAGO score >5 for twelve data sets.

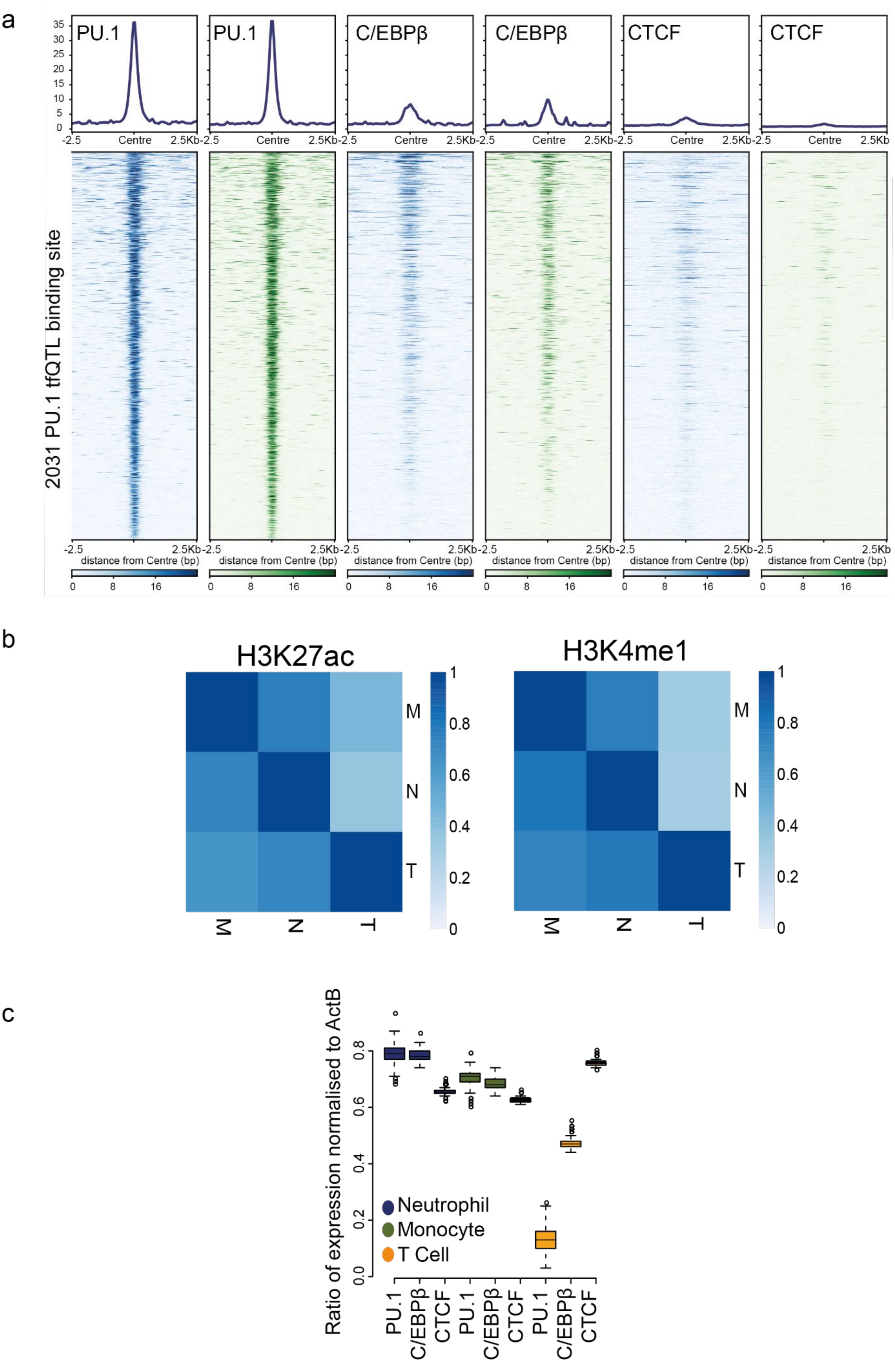

a
PU.1
PU.1
C/EBPβ
C/EBPβ
CTCF
CTCF
35
30
25
20
15
10
5
0
-2.5
Centre
2.5Kb
2031 PU.1 tfQTL binding site
distance from Centre (bp)
0
8
16
b
H3K27ac
H3K4me1
M
N
T
1
0.8
0.6
0.4
0.2
0
c
Ratio of expression normalised to ActB
0.0
0.2
0.4
0.6
0.8
Neutrophil
Monocyte
T Cell
PU.1
C/EBPβ
CTCF
PU.1
C/EBPβ
CTCF
PU.1
C/EBPβ
CTCF

Supplementary Figure 4. Overlap of PU.1 QTLs with chromatin state. a. Sequencing read density heatmap for regions around (+/- 2.5Kb) PU.1 tfQTLs for PU.1, C/EBPβ and CTCF in both neutrophils (blue) and monocytes (green). Percentage of intersecting PU.1 tfQTL peaks which overlap (1bp) with second peak set: PU.1 monocyte 93%, C/EBPβ neutrophil 36%, C/EBPβ monocyte 40%, CTCF neutrophil 11% and CTCF monocyte 5%. **b.** Heatmap of Pi1 statistics of QTL sharing for PU.1 tfQTL across neutrophil, monocyte and T cell types for H3K27ac and H3K4me1 QTLs. **c.** Relative levels of PU.1, C/EBPβ and CTCF gene expression compared to ActB gene expression from ~200 donors across neutrophils, monocytes and CD4⁺ Naïve T cells.

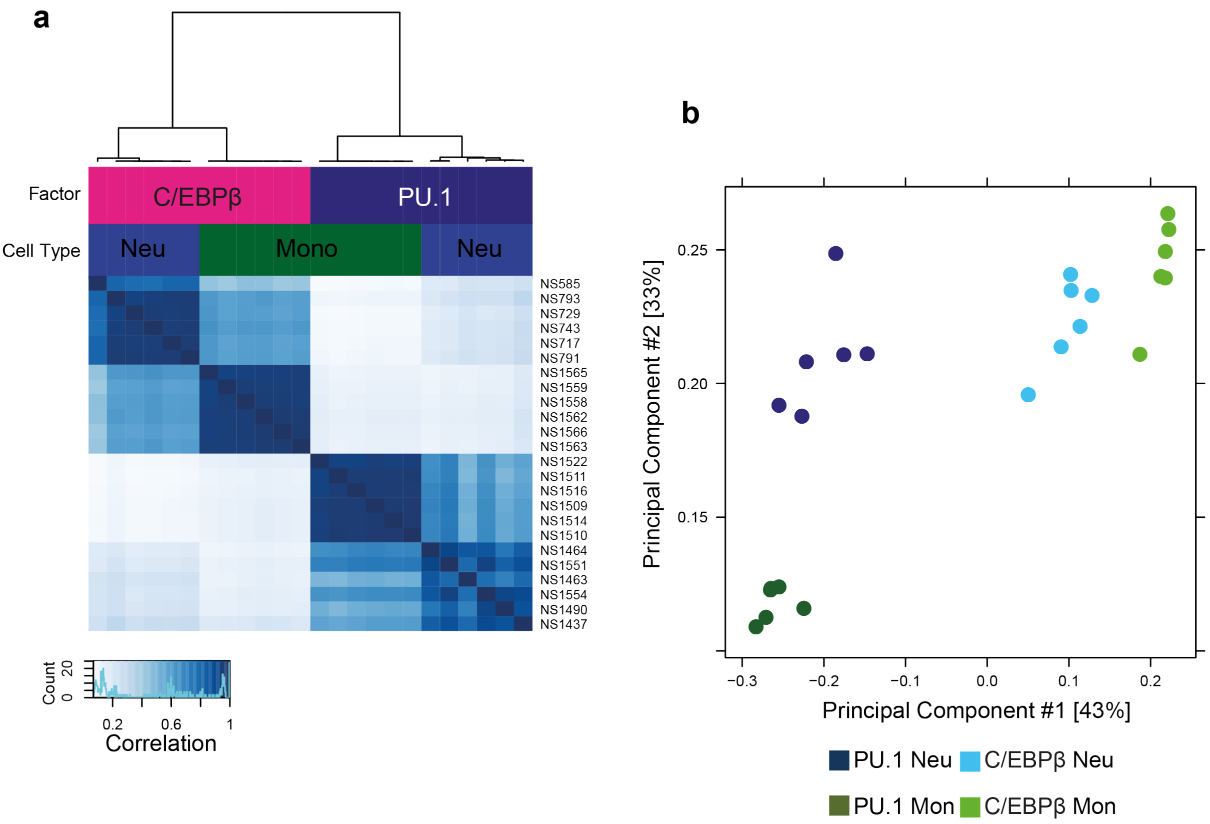

Supplementary Figure 5. Quality control plots of neutrophil and monocyte ChIP-seq datasets that were selected to determine cell type specific transcription factor binding sites. a. Comparison of the data sets selected for differential binding analysis. Heat map showing the correlation of overlaps (1bp) between the peak sets called. **b.** PCA between samples using normalised read counts within consensus peak set.

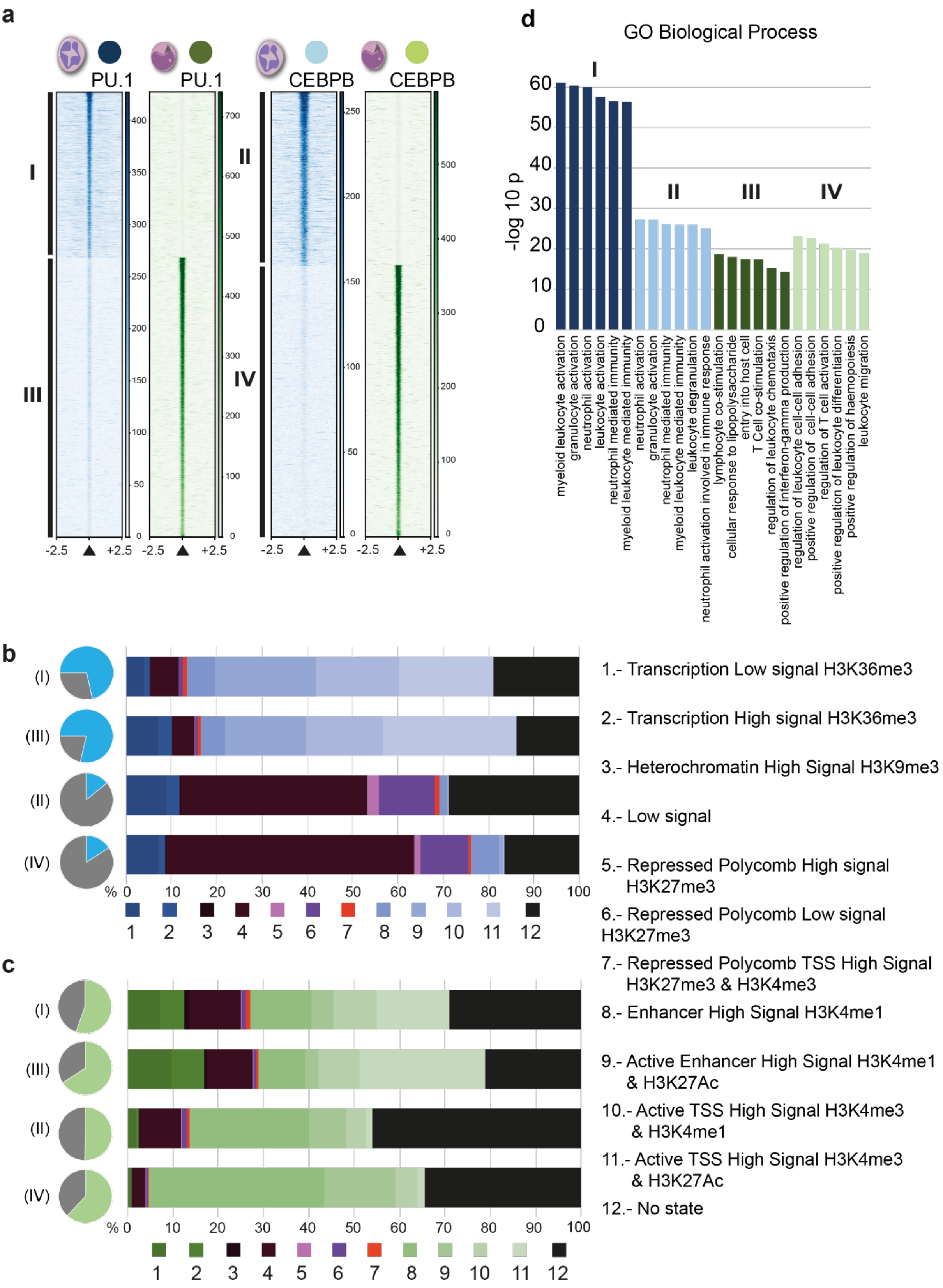

Supplementary Figure 6. Neutrophil PU.1 and C/EBPβ binding sites associate with active chromatin state and genes implicated in myeloid cell processes. a. Pairwise differential binding analysis between Monocytes and Neutrophils for PU.1 and C/EBPβ was performed to identify cell type enriched binding events for the two factors. Read density heatmap +/- 2.5kb from the centre of the binding site. **b.** Intersection of transcription factor binding sites from a. with chromatin state maps for neutrophils derived using ChromHMM [1]. Pie charts; blue segment is the proportion of TF binding

category that fall within regions classed as active in neutrophils. **c.** Intersection of TF binding sites from a. with chromatin state maps for monocytes. Pie charts; green segment is the proportion of TF binding category that fall within regions classed as active in monocytes. **d.** Gene ontology enrichment terms were obtained using GREAT[2] for genes in cis to cell type specific transcription factor binding sites.

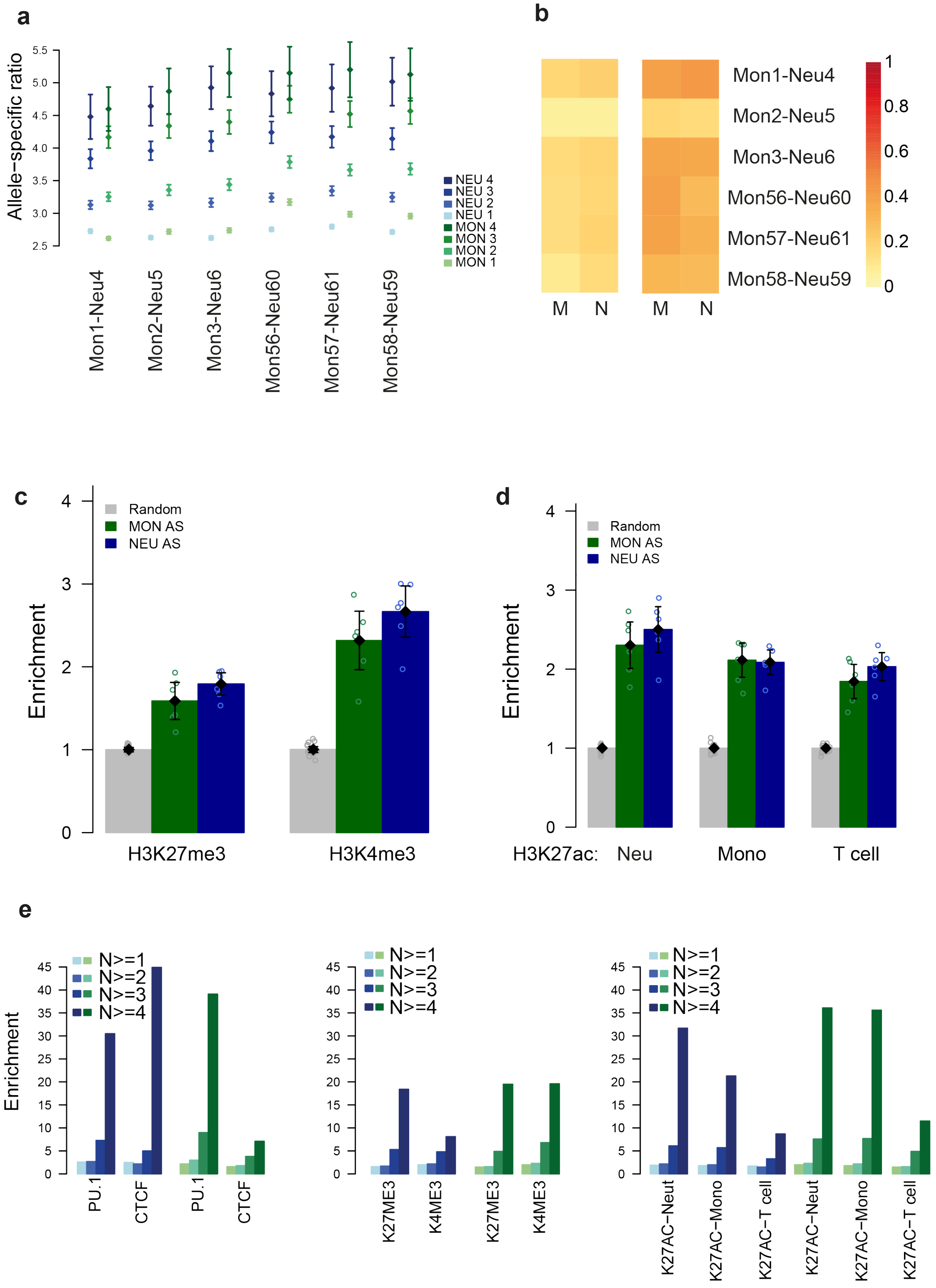

Supplementary Figure 7. Enrichment of tfQTLs at PCHi-C interactions with allelic imbalance. a. Using the PCHi-C data from seven individuals, we selected 310,233 heterozygous sites in neutrophils (NEU) and 288,385 sites in monocytes (MON) with allele-specific (AS) bias >1.5 or <0.67, though we removed sites with extreme Allele Specific (AS) bias (<0.01 or >100). 89% of sites with AS bias in NEU and 92% in MON had a consistent AS ratio if found in more than one individual. The percentage of sites with consistent AS bias detected in two individuals drops to 16% and 15%, and it drops further to just 3% or to <1% when shared by three or four individuals. The plot shows the mean allele-specific (AS) ratio or bias and the 95% confidence interval by individual in neutrophils and monocytes. The mean AS ratio increases when AS ratios are shared across samples in a consistent manner. In almost all individuals AS ratios are higher in monocytes than in neutrophils. **b.** Left heat map; Percentage of sharing between cell types of SNVs that are located in PIRs. Sharing is shown between monocytes (left) and neutrophils (right) in five matched samples (monocytes mean = 14.4%, neutrophils mean = 18.2%) and one mismatched sample (Mon2 and Neu5), (monocytes mean = 6.0%, neutrophils mean = 6.6%). Right heat map; Percentage of sharing between cell types of SNVs that are significant PU.1 QTLs ($p<1x10^{-5}$). Sharing is shown between monocytes (left) and neutrophils (right) in five matched samples (monocytes mean = 37.0%, neutrophils mean = 33.9%) and one mismatched sample (Mon2 and Neu5), (monocytes mean = 17.0%, neutrophils mean = 15.9%). **c.** Enrichment of significant hQTLS (H3K27me3, H3K4me3; Fisher's exact test p<1e-5). The bars represent the mean and the error bars the 95% confidence interval, **d.** Significant hQTLs (H3K27ac; Fisher's exact test p<1e-5) in PIRs. K27AC-Mon represents significant QTLs found in monocytes (linear model p<1e-5) and tested against PIRs found in both monocytes and neutrophils. Similarly, H3K27ac-Neu and H3K27ac-Tcell represent significant QTLs (linear model p<1e-5) found in neutrophils and T-cells and tested against PIRs found in both monocytes and neutrophils. The bars represent the mean and the error bars the 95% confidence interval. **e.** Allele-specific SNVs, identified through PCHi-C, were selected if they were observed in at least two samples or cell types, and if their REF/ALT ratio was consistent, i.e. either > 1 or < 1, across all samples. Enrichment of QTLs in PIRs was then calculated for 14,000 SNPs that fulfilled these criteria and that were supported by an increasing number of samples that showed evidence for falling into a PIR and being a significant QTL (N=1,2,3,4). Enrichment increased when increasing the number of supporting samples.

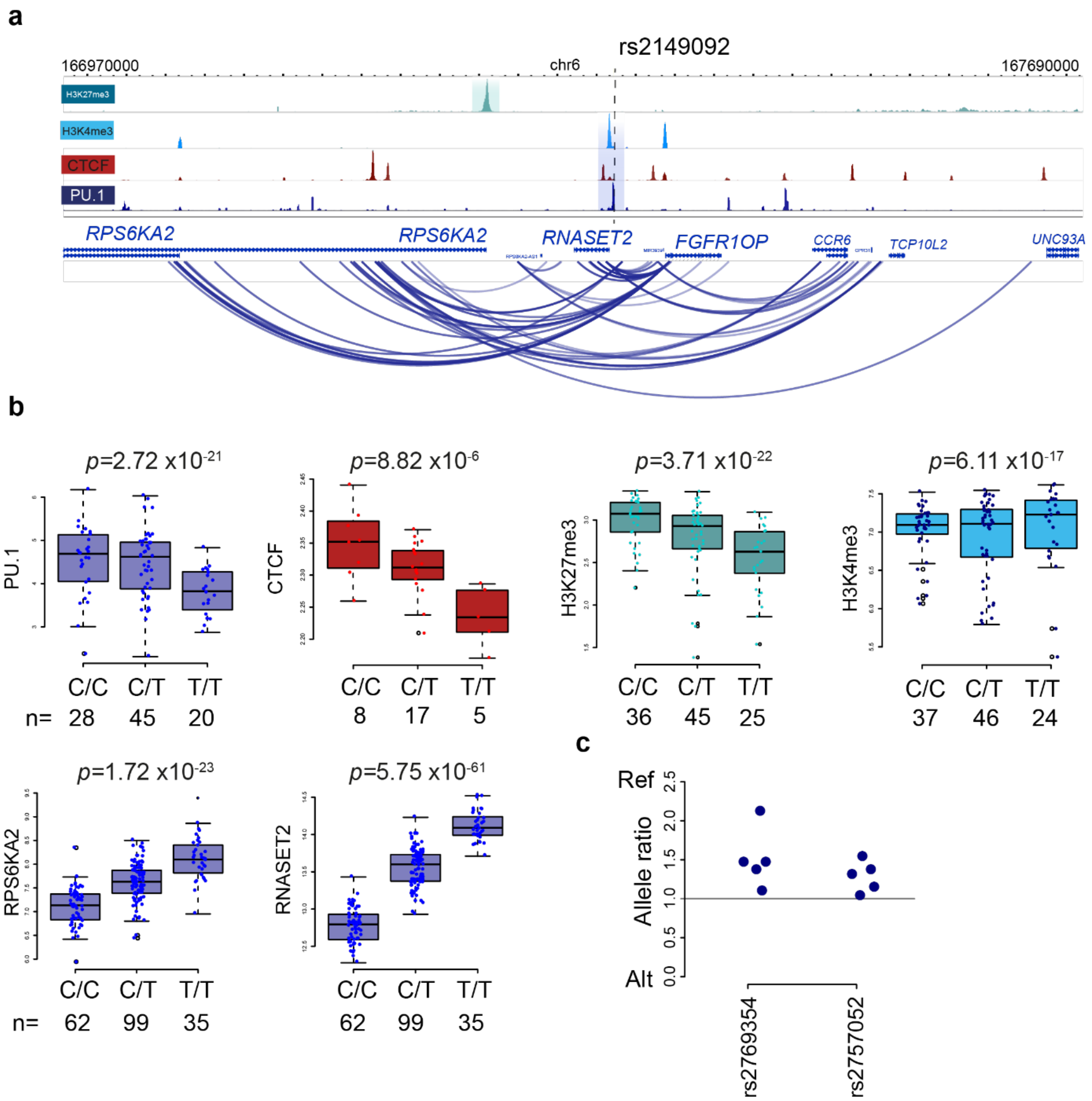

Supplementary Figure 8. Example loci of a repressive PU.1 tfQTL associated with autoimmune disease a. Genome browser view at the *RNASET2* / *RPS6KA2* locus displaying illustrative ChIP enrichment tracks for H3K27me3 (cadet blue), H3K4me3 (deep sky blue), PU.1 (navy) and CTCF (fire brick red) in neutrophils. The lead SNP, rs2149092 is highlighted by a dashed line. Associated tfQTL and hQTL regions are highlighted by shaded regions. Beneath, PCHi-C interaction data from neutrophils (CHiCAGO score >5). **b.** Signal boxplots for significant tfQTL, hQTL and eQTL segregated by the lead SNP rs2149092, (C is the reference allele). Box plots show the medians (centre lines) and the twenty-fifth and seventy-fifth percentiles (box edges), with whiskers extending to 1.5 times the interquartile range. *p*-value were obtained by fitting linear mixed models implemented in LIMIX. n= the number of individual donors. **c.** rs2769354 and rs2757052 are tag SNPs for the lead variant ([LD] $r^{2=}$0.97) which were assayed in the allele specific PCHi-C analysis. 5 of the 6 donors are heterozygous. Ratio is the number of adjusted PCHi-C reads aligning to reference allele versus the alternate allele.

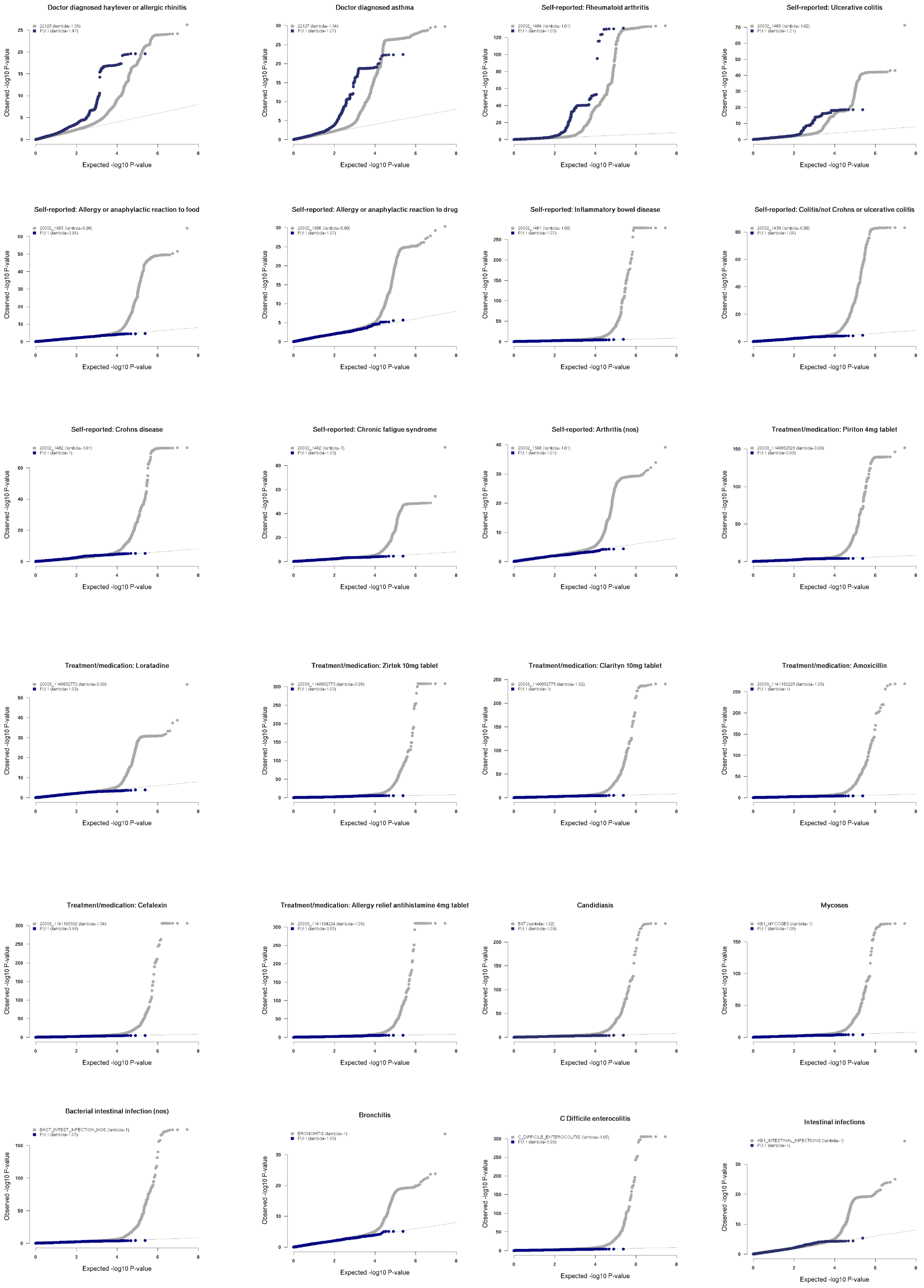

Supplementary Figure 9. Enrichment of PU.1 tfQTL SNPs within GWAS summary statistics from traits collected as part of the UK Biobank study. Quantile-quantile plots from 24 GWAS traits associated with allergies, autoimmune disease and infection.

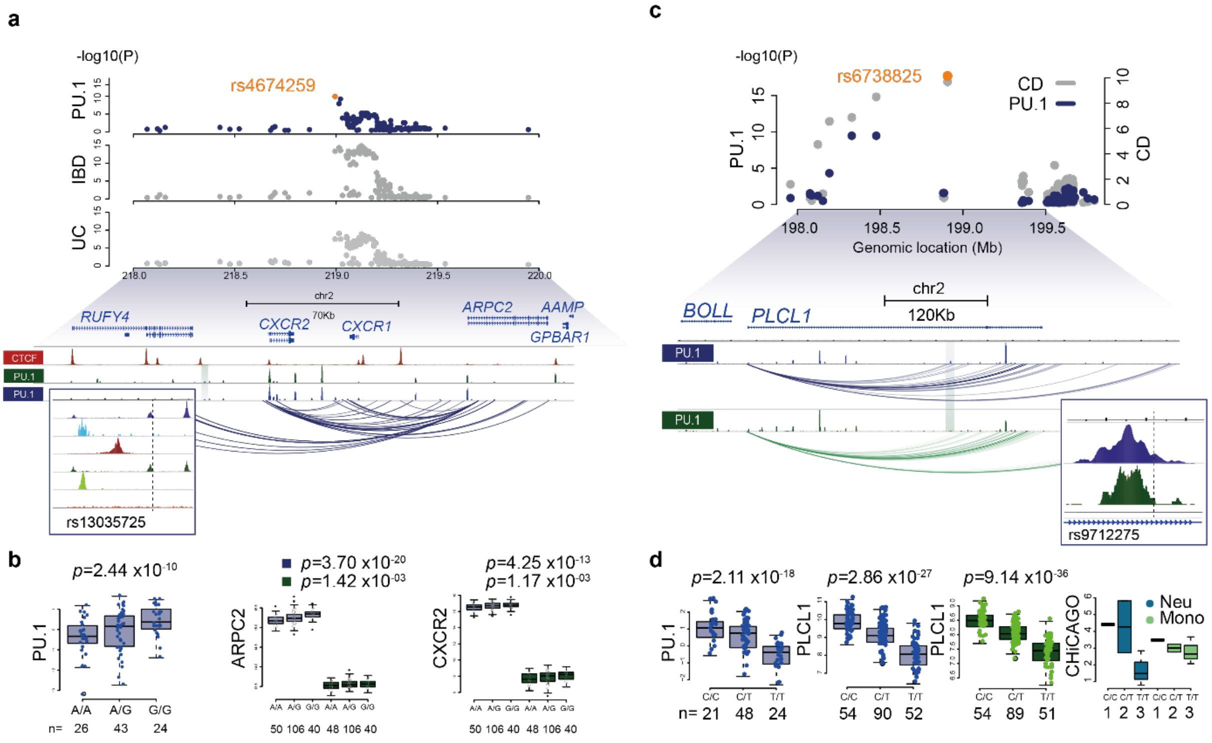

Supplementary Figure 10. Examples of disease associated loci. a. Example of colocalised signal for PU.1 tfQTL, rs13035725 (p = 2.44x10^{-10}) identifies a risk locus for inflammatory bowel disease and ulcerative colitis. Locus zoom plot depicting -log10(P) values for PU.1 tfQTL, IBD and UC associations. In orange location of rs4674259 the most significant shared SNP between tfQTL and GWAS data sets. Genome browser view of locus displaying ChIP-seq data for PU.1 in neutrophils (navy) and monocytes (dark olive) transcription factor binding sites. Inset; zoom of region displaying ChIP-seq for PU.1 in neutrophils (navy) and monocytes (dark olive). C/EBPβ neutrophils (light blue) and monocytes (light olive). CTCF neutrophils (firebrick) and monocytes (red). The region also displayed high connectivity in the neutrophil PCHi-C data (CHiCAGO score > 5), but depleted of significant interactions in monocytes. The same SNP is also significantly associated with expression of several genes in neutrophils, including *ARPC2* (p=3.70x10^{-20}) and *CXCR2* (p=4.25x10^{-13}). **b.** Signal boxplots for PU.1 in neutrophils, gene expression for *ARPC2* and *CXCR2* segregated by genotype for lead SNP rs13035725, neutrophils (navy) and monocytes (dark olive). Box plots show the medians (centre lines) and the twenty-fifth and seventy-fifth percentiles (box edges), with whiskers extending to 1.5 times the interquartile range. p-value were obtained by fitting linear mixed models implemented in LIMIX. n= number of individual donors. **c.** Example of colocalised signal for PU.1 tfQTL rs9712275 with Crohn's disease. Manhattan plot of the -log10(P) values for shared SNPs from PU.1 tfQTL (navy) and Crohn's disease (grey), position of lead shared SNP rs6738825 highlighted in orange. Genome browser shot of PU.1 binding and promoter interacting regions baited to *PLCL1* gene both neutrophils (navy) and monocytes (green). Lead PU.1 QTL peak is highlighted by shaded area. Inset; zoom of ChIP-seq data for PU.1 in neutrophils (navy) and monocytes (dark olive), dashed line indicates position of lead SNP. **d.** Boxplots of signals for molecular traits; PU.1, gene expression and PCHi-C split by donor genotype for rs9712275. Box plots show the medians (centre lines) and the twenty-fifth and seventy-fifth percentiles (box edges), with whiskers extending to 1.5 times the interquartile range. p-value were obtained by fitting linear mixed models implemented in LIMIX. n= number of individual donors.

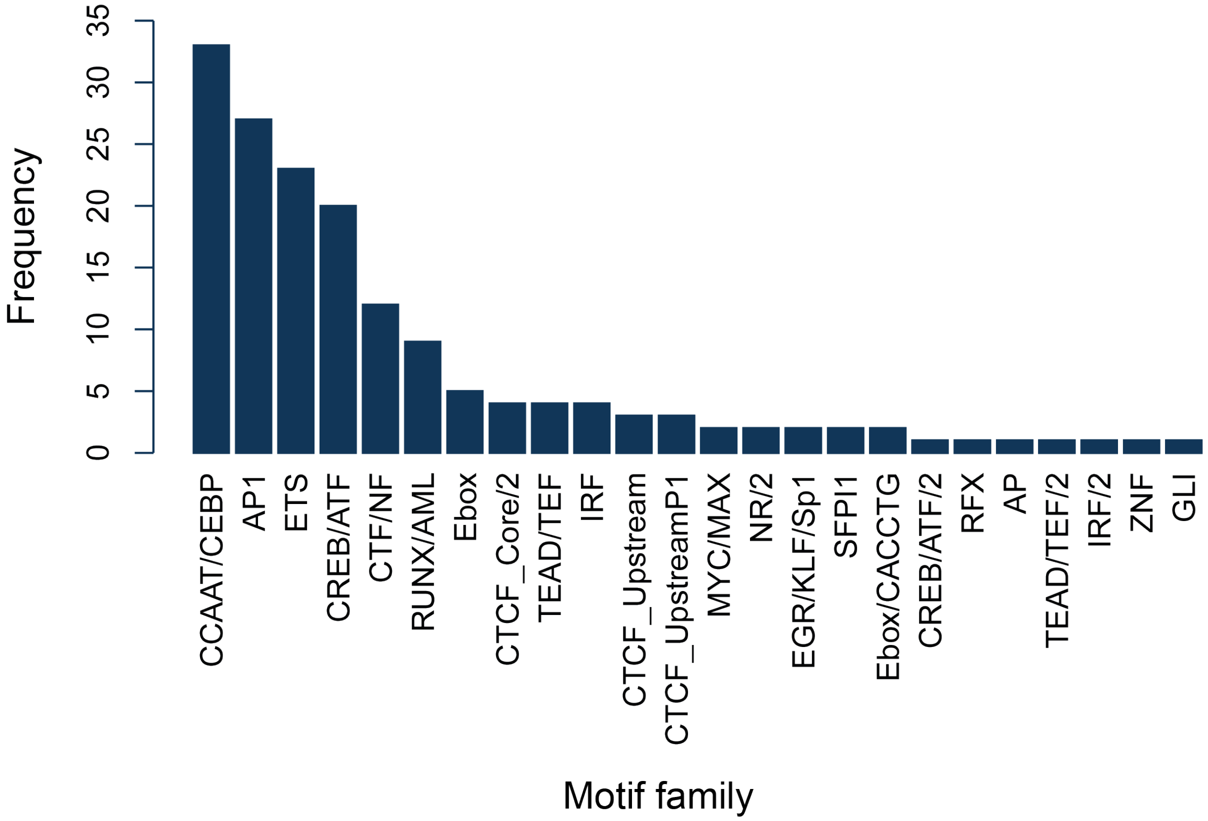

Supplementary Figure 11. Frequency of motif disruption for transcription factor families at colocalised loci. Bar plot with the frequency of motif families with a predicted transcription factor disruption (CATO score >0.1). The top 6 clusters harbour 79% of the 231 tfQTLs (*i.e.* PU.1 lead tfQTLs and proxies with LD>0.8).

Supplementary References

1. Carrillo-de-Santa-Pau, E. *et al.* Automatic identification of informative regions with epigenomic changes associated to hematopoiesis. *Nucleic Acids Res* **45**, 9244-9259 (2017).
2. McLean, C.Y. *et al.* GREAT improves functional interpretation of cis-regulatory regions. *Nat Biotechnol* **28**, 495-501 (2010).
