# Supplementary material for: Genetic perturbation of PU.1 binding and chromatin looping at neutrophil enhancers associates with autoimmune disease
Source: Nat Commun. 2021 Apr 16;12:2298. doi: 10.1038/s41467-021-22548-8 (PMC8052402; doi:10.1038/s41467-021-22548-8)
Supplement: Supplementary file 3 — Description of Additional Supplementary Files [file 41467_2021_22548_MOESM3_ESM.pdf]

Supplementary Data

Supplementary Data 1. ChIP-seq data, enrichment and quality control metrics. ChIP-seq quality control metrics including number of aligned reads, percent duplicate reads, MACS peaks called. Including metadata for sample identification, antibody and gender.

Supplementary Data 2. Quantitative trait loci results. Results from Limix QTL analysis for each feature tested for PU.1, C/EBPβ, CTCF, H3K4me3 and H3K27me3 in neutrophils and the statistical test results for each sentinel SNP.

Supplementary Data 3. Proximal and distal quantitative trait loci results for sentinel PU.1 SNPs. Classification of proximal and distal PU.1 tfQTLs reaching the significance threshold (FDR<0.05).

Supplementary Data 4. PU1 tfQTLs that overlap autoimmune diseases and UK Biobank myeloid traits. Summary results from colocalisation analysis for PU.1 tfQTL with selected disease and full blood count traits.

Supplementary Data 5. Annotation of PU1 tfQTLs. PU.1 tfQTL were annotated for significant allele-specific effect (RASQUAL analysis), neutrophil gene expression QTL, baited genes through PCHi-C interaction data and whether QTL summary statistics colocalise with any of the GWAS traits tested.
