# Supplementary material for: Genetic perturbation of PU.1 binding and chromatin looping at neutrophil enhancers associates with autoimmune disease
Source: Nat Commun. 2021 Apr 16;12:2298. doi: 10.1038/s41467-021-22548-8 (PMC8052402; doi:10.1038/s41467-021-22548-8)
Supplement: Supplementary file 9 — Reporting Summary [file 41467_2021_22548_MOESM9_ESM.pdf]

nature research

Corresponding author(s): Prof. Nicole Soranzo

Last updated by author(s): Feb 22, 2021

Reporting Summary

Nature Research wishes to improve the reproducibility of the work that we publish. This form provides structure for consistency and transparency in reporting. For further information on Nature Research policies, see our Editorial Policies and the Editorial Policy Checklist.

Statistics

For all statistical analyses, confirm that the following items are present in the figure legend, table legend, main text, or Methods section.

n/a	Confirmed	
☐	☒	The exact sample size (*n*) for each experimental group/condition, given as a discrete number and unit of measurement
☐	☒	A statement on whether measurements were taken from distinct samples or whether the same sample was measured repeatedly
☐	☒	The statistical test(s) used AND whether they are one- or two-sided<br>*Only common tests should be described solely by name; describe more complex techniques in the Methods section.*
☐	☒	A description of all covariates tested
☐	☒	A description of any assumptions or corrections, such as tests of normality and adjustment for multiple comparisons
☐	☒	A full description of the statistical parameters including central tendency (e.g. means) or other basic estimates (e.g. regression coefficient) AND variation (e.g. standard deviation) or associated estimates of uncertainty (e.g. confidence intervals)
☐	☒	For null hypothesis testing, the test statistic (e.g. *F*, *t*, *r*) with confidence intervals, effect sizes, degrees of freedom and *P* value noted<br>*Give P values as exact values whenever suitable.*
☒	☐	For Bayesian analysis, information on the choice of priors and Markov chain Monte Carlo settings
☒	☐	For hierarchical and complex designs, identification of the appropriate level for tests and full reporting of outcomes
☐	☒	Estimates of effect sizes (e.g. Cohen's *d*, Pearson's *r*), indicating how they were calculated

Our web collection on statistics for biologists contains articles on many of the points above.

Software and code

Policy information about availability of computer code

Data collection: No Software

Data analysis:
R 3.4.0.
Limix https://github.com/PMBio/limix .
GATK ASEReadCounter (ver3.4)
Rasqual https://github.com/natsuhiko/rasqual
BEAGLE2 (v2.0.5)
GARFIELD
plink (v1.9)
regioneR (1.0.3).
Picard MarkDuplicates (v1.103).
SAMtools (v0.1.18)
PhantomPeakQualTools (version18)
MACS (v2.0.10.20131216)
BEDOPS (v2.4.14)
DiffBind (version 1.12.0)
EdgeR (3.8.3)
HiCUP (v0.5.5)
Chicago (v0.2.5)
Deeptools 2(Galaxy Version 3.3.2.0.1)
BWA (v0.5.9)
WASP

For manuscripts utilizing custom algorithms or software that are central to the research but not yet described in published literature, software must be made available to editors and reviewers. We strongly encourage code deposition in a community repository (e.g. GitHub). See the Nature Research guidelines for submitting code & software for further information.

Data

Policy information about availability of data

All manuscripts must include a data availability statement. This statement should provide the following information, where applicable:

- Accession codes, unique identifiers, or web links for publicly available datasets
- A list of figures that have associated raw data
- A description of any restrictions on data availability

Data links to sequence files:
https://www.ebi.ac.uk/ega/datasets/EGAD00001004571
https://www.ebi.ac.uk/ega/datasets/EGAD00001002711
https://www.ebi.ac.uk/ega/datasets/EGAD00001002712
https://www.ebi.ac.uk/ega/studies/EGAS00001001911

Access to the sequence and alignment and genotype level data produced by the Blueprint Consortium is controlled by the Blueprint Data Access Committee (DAC). Access to data will be granted to qualified investigators for appropriate use. Please follow the link http://www.blueprint-epigenome.eu/index.cfm?p=B5E93EE0-09E2-5736-A708817C27EF2DB7 for the application form and data access aggrement. Please send any questions to blueprint-dac@ebi.ac.uk

Access to PU.1, CEBPB, CTCF associated DAC agreement, Wellcome Trust Sanger Institute, Access information: http://www.ebi.ac.uk/ega/dacs/EGAC00001000205, Contact Person: Data Sharing, Email: datasharing@sanger.ac.uk

Data from UK Biobank GWAS graciously provided by the Neale lab. Available at: http://www.nealelab.is/uk-biobank . (release UKBB GWAS Imputed v3 - File Manifest Release 20180731)

Field-specific reporting

Please select the one below that is the best fit for your research. If you are not sure, read the appropriate sections before making your selection.

☒ Life sciences ☐ Behavioural & social sciences ☐ Ecological, evolutionary & environmental sciences

For a reference copy of the document with all sections, see nature.com/documents/nr-reporting-summary-flat.pdf

Life sciences study design

All studies must disclose on these points even when the disclosure is negative.

Sample size

138 study participants. No sample size calculation was performed, with previous studies (such as Waszak et al., Cell 2015) demonstrating that studies with even much smaller numbers of donors can reliably detect some molecular QTLs. Increasing the number of donors increases the power of QTL detection, so here we selected a maximum feasible number of donors given logistical and resource constraints.”.

Data exclusions

Donors: S00HE8
S00G7Q
S00E9U
S00G5U
S00GEC
S00J3M
S00DQ0
S00Q00
S00DKC
S00G9M
S00FUL
S00FVJ
S00F9Q
S00GSL
S00HUD
S00D9Y
S00DP2
S00GWD
S00FK4
S00FPV
S00GBI

	S00HVB<br>S00QY6<br>S00P6T<br>S00N36<br>for H3K4me3 and H3K27me3 are present in the EGA data release but were not included in the study.
Replication	For ChIP-seq experiments there was a minimum of 22 independent donors for each factor assayed. Enabling statistical confidence for common genetic variants MAF>5. For PU.1 there three technical replicates performed on the same donor. For PCH-C there were 6 independent donors for both cell types. Each sample contributed individually to QTL detection as is standard practice. Reference sets were based on all samples combined.
Randomization	Random allocation
Blinding	Donors were provided with anonymised identifiers, labels were not blinded as this approach is not appropriate for QTL detection

Reporting for specific materials, systems and methods

We require information from authors about some types of materials, experimental systems and methods used in many studies. Here, indicate whether each material, system or method listed is relevant to your study. If you are not sure if a list item applies to your research, read the appropriate section before selecting a response.

Materials & experimental systems

n/a	Involved in the study
☐	☒ Antibodies
☒	☐ Eukaryotic cell lines
☒	☐ Palaeontology and archaeology
☒	☐ Animals and other organisms
☐	☒ Human research participants
☒	☐ Clinical data
☒	☐ Dual use research of concern

Methods

n/a	Involved in the study
☐	☒ ChIP-seq
☒	☐ Flow cytometry
☒	☐ MRI-based neuroimaging

Antibodies

Antibodies used	Antibodies for H3K4me3 (C15410003 lot A5051-001D), H3K27me3 (C15410195 lot A1811-001D), CTCF (C15410210 lot A2359-0010) were obtained from Diagenode, Liege, Belgium. Antibodies for PU.1 (sc-352x lot B2415, sc-22805x lot D0609) and C/EBPβ (sc-150x lot G1814) were obtained from Santa Cruz Biotechnology.
Validation	http://www.blueprint-epigenome.eu/UserFiles/File/Protocols/BLUEPRINT_ANTIBODIES_VALIDATION_SOP___2_.pdf<br>Blueprint validated antibodies have been extensively used in publications including Yi et al; Cell Reports 2019<br>http://datasheets.scbt.com/sc-352.pdf and has been extensively used in publications including Heniz et al; Nature 2013<br>http://datasheets.scbt.com/sc-150.pdf and has been extensively used in publications including Link et al; Cell 2018<br>http://datasheets.scbt.com/sc-22805 and has been extensively used in publication including Kilpinen et al; Science 2013

Human research participants

Policy information about studies involving human research participants

Population characteristics	Donors were on average 55 years old (range 20-75 years old), with 46% of donors being male. A full blood count (FBC) for all donors was obtained from an EDTA blood sample, collected in parallel with the whole blood unit, using a Sysmex Haematological analyser. The level of C-reactive protein (CRP), an inflammatory marker, was also measured in the sera of all individuals. All donors used for the collection had FBC and CRP parameters within the normal healthy range.
Recruitment	Blood was obtained from donors who were members of the NIHR Cambridge BioResource (http://www.cambridgebioresource.org.uk/) with informed consent at the NHS Blood and Transplant, Cambridge, UK. Participants were blood donors who provide for the UK NHS Blood and transplant service who agreed to take part in the study, there was no pre-selection criteria.
Ethics oversight	NRES Committee East of England - Hertfordshire<br>Study title:A Blueprint of Blood Cells<br>REC reference:12/EE/0040

Note that full information on the approval of the study protocol must also be provided in the manuscript.

ChIP-seq

Data deposition

☒ Confirm that both raw and final processed data have been deposited in a public database such as GEO.

☒ Confirm that you have deposited or provided access to graph files (e.g. BED files) for the called peaks.

Data access links<br>*May remain private before publication.*	https://www.ebi.ac.uk/ega/datasets/EGAD00001004571 (H3K4me3)<br>https://www.ebi.ac.uk/ega/datasets/EGAD00001002711 (H3K27me3)<br>https://www.ebi.ac.uk/ega/datasets/EGAD00001002712 (TF)<br>https://www.ebi.ac.uk/ega/studies/EGAS00001001911 (PCHi-C)
Files in database submission	Aligned .BAM files and peaks.
Genome browser session<br>(e.g. UCSC)	http://genome.ucsc.edu/s/steve9110/NatComms_sbw

Methodology

Replicates	93 independent Primary neutrophil PU.1 plus 3 technical replicates from the same donor generated on different days. neutrophil samples from partially overlapping donors 22 CEBPB, 30 CTCF, 107 H3K4me3, 106 H3K27me3. Monocyte donor samples 10 PU.1, 9 CEBPB & 4 CTCF.
Sequencing depth	We aimed for a minimum of 10 million QC passed reads for narrow marks and 30 million QC passed reads for broad marks. Full details in Supplementary Table 1.
Antibodies	Antibodies for H3K4me3 (C15410003), H3K27me3 (C15410195), CTCF (C15410210) were obtained from Diagenode, Liege, Belgium. Antibodies for PU.1 (sc-352x, sc-22805x) and C/EBPβ (sc-150x) were obtained from Santa Cruz Biotechnology.
Peak calling parameters	MACS2 (v2.0.10.20131216, standard options) for peak calling with the estimated fragment size from PhantomPeakQualTools (--shiftsize=half fragment size), with narrow for PU.1, C/EBPβ, CTCF, H3K4me3 and broad flags set for H3K27me3. For background control ChIP input was created from merging random selected samples. Significant peaks were selected to be at 1% FDR or less.
Data quality	We aimed for a minimum of 10 million QC passed reads for narrow marks and 30 million QC passed reads for broad marks. We aimed for a minimum of 10,000 peaks to be called for each data set. We removed ChIP samples that had a relative strand correlation (RSC) < 0.8 and normalised strand correlation (NSC) < 1.05 61. We defined high confidence data those from ChIP with RSC > 0.8 and NSC > 1.05. Otherwise, we used genome browser tracks to confirm visually a good ChIP and include it in the final data set.
Software	Sequenced reads were aligned to reference genome using BWA (bwa aln –q 15). Duplicate reads were marked using Picard MarkDuplicates (v1.103). Reads with mapping quality less than 15 were removed (SAMtools v0.1.18). The fragment size L for each aligned bam was estimated using PhantomPeakQualTools vr18, which uses cross correlation of binned read counts between forward and reverse strands. To identify highly enriched genomic regions, we used MACS2 (v2.0.10.20131216, standard options) for peak calling with the estimated fragment size from PhantomPeakQualTools (--shiftsize=half fragment size), with narrow for PU.1, C/EBPβ, CTCF, H3K4me3 and broad flags set for H3K27me3. Consensus peak sets were constructed using dba.peakset function within DiffBind R package (1.12.0).
